# Predictors of Nonsentinel Nodal Involvement to Aid Intraoperative Decision Making in Breast Cancer Patients with Positive Sentinel Lymph Nodes

**DOI:** 10.5402/2011/539503

**Published:** 2011-08-16

**Authors:** Ern Yu Tan, Bernard Ho, Juliana J. C. Chen, Pey Woei Ho, Christine Teo, Arul Earnest, Patrick M. Y. Chan

**Affiliations:** ^1^Department of General Surgery, Tan Tock Seng Hospital, 11 Jalan Tan Tock Seng, Singapore 308433; ^2^Department of Pathology, Tan Tock Seng Hospital, 11 Jalan Tan Tock Seng, Singapore 308433; ^3^Centre for Quantitative Medicine, Duke-NUS Graduate Medical School Singapore, 8 College Road, Singapore 169857

## Abstract

*Background*. Up to 60% of patients with a positive sentinel lymph node (SLN) have no additional nodal involvement and do not benefit from completion axillary lymph node dissection (ALND). We aim to identify factors predicting for non-SLN involvement and to validate the MSKCC nomogram and Tenon score in our population. *Methods*. Retrospective review was performed of 110 consecutive patients with positive SLNs who underwent ALND over an 8-year period. *Results*. Fifty patients (45%) had non-SLN involvement. Non-SLN involvement correlated positively with the number of positive SLNs (*P* = 0.04), macrometastasis (*P* = 0.01), and inversely with the total number of SLNs harvested (*P* = 0.03). The MSKCC nomogram and Tenon score both failed to perform as previously reported. *Conclusions*. The MSKCC nomogram and Tenon score have limited value in our practice. Instead, we identified three independent predictors, which are more relevant in guiding the intraoperative decision for ALND.

## 1. Introduction

Sentinel lymph node (SLN) biopsy is the current standard of care for T1 T2 breast cancers with no clinically palpable axillary lymph nodes. Based on the principle of sequential directional lymphatic drainage from the breast, the SLN is hypothetically the first axillary node to receive lymphatic drainage from the breast. It, therefore, follows that if the SLN is free of metastatic tumour deposits, the rest of the axillary nodes are not expected to be involved either. This obliterates the need for full axillary lymph node dissection (ALND) when the SLN is negative for metastases. ALND, involving the removal of the level I and II axillary nodes, is now reserved for instances where the SLN is positive for metastases or where SLN biopsy is contraindicated. This has resulted in more than 50% of patients with T1 and T2 tumours being spared the arm morbidity of ALND because of the less extensive dissection [1, 2].

However, it is known that up to 60% of patients with a positive SLN do not have additional nodal involvement, suggesting that these patients are overtreated by the current practice of ALND whenever the SLN is positive [3, 4]. The removal of uninvolved nodes serves neither to aid prognostication nor to guide adjuvant therapy, yet exposes the patient to the risk of lymphedema and its associated complications. This has led to some questioning the need for ALND in all instances of SLN positivity [5]. The American College of Surgeons Oncology Group Z0011 trial is the latest to show that women with T1 and T2 tumours who undergo lumpectomy derive little additional benefit from ALND since any residual disease in the level I and II nodes appear to be effectively eradicated by postoperative irradiation and chemotherapy [6, 7]. It remains to be seen whether the evidence so far will change current guidelines. Most centres continue to advocate ALND when the SLN is positive. Given the reluctance of most surgeons to leave behind residual disease in the axilla, a reliable means of predicting the likelihood of non-SLN involvement will be a step towards refining the indication for ALND. To date, there are 4 nomograms (Memorial Sloan-Kettering Cancer Centre [MSKCC] nomogram [8], Mayo nomogram [9], Cambridge nomogram [10] and the Stanford nomogram [11]), 3 scoring systems (the Tenon score [12], the MD Anderson Cancer Centre score [13] and the score developed by Saidi et al. [14]) and 2 recursive partitioning tools developed by Kohrt et al. [11]. In a direct comparison of these models, the MSKCC nomogram and the Tenon score performed best and were the only 2 models with an area under the curve (AUC) of more than 0.75 [15]. 

In our practice, the decision for ALND is made intraoperatively based on frozen section analysis of the SLN. Apart from guiding the decision to proceed with ALND during surgery, a reliable means of predicting the status of the non-SLN nodes will also reduce patient recall rates should the initial frozen section analysis be false negative. We, therefore, aim to determine the incidence of non-SLN involvement in our patients, and to identify factors that may predict for this. In addition, we aim to validate the MSKCC nomogram and the Tenon score in our local population.

## 2. Methods

A retrospective review was performed of 110 consecutive patients with a positive SLN who underwent ALND from 1st January 2001 to 31st December 2008 in our department. This study has ethics committee approval (DSRB D/10/029). In our department, the majority of SLN biopsy is performed with blue dye alone. Two mL of undiluted patent V blue dye is injected into the subareolar plexus after the patient is put under general anaesthesia, followed by 5 minutes of manual massage. The SLN is identified as a blue-coloured node with a blue lymphatic channel leading up to it. Beginning from the year 2006, all SLNs were routinely submitted for intraoperative frozen section analysis (a total of 64 patients). This involves the documentation of the number and size of SLNs submitted for analysis, followed by histological examination of serial sections stained with haematoxylin and eosin (H&E). Each SLN is serially sliced at 2 to 3 mm gross intervals, and all slices (the entire node) are snap frozen in liquid nitrogen and then placed in frozen section embedding medium on a cryostat object disk. Each slice is then further sectioned at intervals of 40 *μ*m to obtain at least 3 sections, which are stained in H&E and examined using routine light microscopy. The presence or absence of metastatic deposits is noted and communicated to the surgeon immediately. If the SLN is positive for macro- or micrometastasis, ALND is immediately proceeded with; if negative, no further axillary dissection is performed. The entire SLN is then formalin fixed and paraffin embedded to obtain permanent sections for final analysis, where an additional 1 to 6 levels of each slice of the SLN are examined. It is not routine in our practice to perform immunohistochemistry or quantitative real-time polymerase chain reactions on H&E-negative sections. For the 46 patients who underwent surgery prior to 2006, only serial H&E analysis of the permanent sections of the SLN was performed (without frozen section analysis).

The presence of metastatic deposits in the SLN and non-SLNs was correlated with standard clinicopathological parameters including histology of the primary tumour, tumour size, tumour grade, presence of lymphovascular invasion, and hormone and human epidermal growth factor receptor 2 (HER2) receptor status. Note was also made of whether the metastatic deposit was classified as a macrometastasis or micrometastasis. Macrometastases are defined as cell clusters that are more than 2 mm in diameter; micrometastatic deposits are defined as cell clusters that are between 0.2 mm and 2 mm in diameter (denoted as N1_mic_ according to the 6th AJCC classification). Correlation analyses were performed using either the chi square test or Fisher's exact test where appropriate. Correlation with tumour grade was evaluated using the Chi square test for trend. All univariate analyses were performed with GraphPadPrism version 4 (GraphPad software Inc., San Diego CA). The Cox proportional hazard regression model was used to identify independent risk factors for non-SLN involvement. This was carried out using the Stata package release 8.1 (Stata Corporation, 4905 Lakeway Drive, College Station, Texas 77845, USA). A full model was first created to include all potentially important explanatory variables. At each step, the variable with the smallest contribution to the model was removed, until a final backward stepwise model was obtained. A 2-tailed *P* value test was used in all analyses and a *P* value of less than 0.05 was considered statistically significant. 

The probability of non-SLN involvement was calculated based on the MSKCC nomogram and Tenon score. The MSKCC nomogram is based on tumour histology and grade, pathological tumour size, multifocality, lymphovascular invasion, oestrogen receptor (ER) status, the number of positive and negative SLNs, and the mode of detection (in our study, SLN biopsy cases prior to 2006 were performed with serial H&E examination of the permanent sections, and those from 2006 onwards were assessed with intraoperative frozen section analysis). The combined score of each variable is translated into a predicted probability of non-SLN involvement. The nomogram is available as an electronic calculator on the MSKCC website (http://www.mskcc.org/mskcc/html/15938.cfm). The Tenon score is calculated based on three variables: the ratio of positive SLN to the total number of SLN removed, the presence of micrometastasis in the SLN, and the primary tumour size, combined to give a score from 0 to 7 [12]. Receiver operating characteristic (ROC) curves based on both models were generated and the area under ROC curve (AUC) calculated using Stata package release 8.1. AUC ranged from 0 to 1, where 1 indicates perfect concordance, 0.5 indicates no better concordance than chance, and 0 indicates perfect disconcordance.

## 3. Results

From 1st January 2001 to 31st December 2008, a total of 551 patients underwent SLNB in our centre; of these, 110 patients underwent completion ALND after a positive SLN was found. Median patient age was 53 years (30 to 80 years). All patients were diagnosed with invasive carcinoma; 88% (97 of 110) were classified as invasive ductal carcinoma, 10% as invasive lobular carcinoma. One patient had a medullary carcinoma and another a mucinous carcinoma. Median pathological tumour size was 21 mm (4 mm to 80 mm), and median tumour grade was 2. Seventy percent of patients had oestrogen receptor (ER) positive tumours, and 47% had progesterone receptor (PR) positive tumours. The median number of SLN harvested was 2 (1 to 9). All except 9 SLN biopsies were performed using blue dye alone, 6 were performed using dual blue dye and radiocolloid, and 3 were performed with radiocolloid alone. Median number of axillary lymph nodes harvested (including SLNs) was 22 (ranging from 7 to 58).

The SLN was the only positive axillary node in 60 of 110 patients (55%). The likelihood of non-SLN involvement correlated positively with pathological tumour size (*P* = 0.03); median tumour size was 24.5 mm in cases of non-SLN involvement compared to 20 mm in those where only the SLN was involved. Non-SLN involvement was also inversely correlated with the ratio of positive SLNs to the total number of SLNs harvested (*P* = 0.01) (Table 1). Those with a ratio of 0.5 or more were 3 times more likely to have non-SLN involvement (*P* = 0.04, OR 2.63, 95% CI 1.04 to 6.64), implying that additional non-SLN involvement became less likely when more harvested SLNs were negative. The size of the metastatic deposits also correlated with the likelihood of non-SLN involvement, with macrometastasis (rather than micrometastasis) being associated with involvement of the non-SLNs (*P* = 0.01, OR = 14.9, 95% CI 0.01 to 0.53) (Table 1). Non-SLN involvement did not correlate with tumour histology, tumour grade, lymphovascular invasion, or hormone receptor status (*P* > 0.05) (Table 1). Non-SLN involvement also did not increase the likelihood of distant recurrence. On multivariate analysis, the number of positive SLNs, total number of SLNs harvested, presence of micrometastasis, and the total number of axillary nodes harvested during ALND independently predicted for non-SLN involvement (*P* < 0.05) (Table 2). Interestingly, tumour size did not remain significant (*P* = 0.14) (Table 2). 

**Figure 1:**
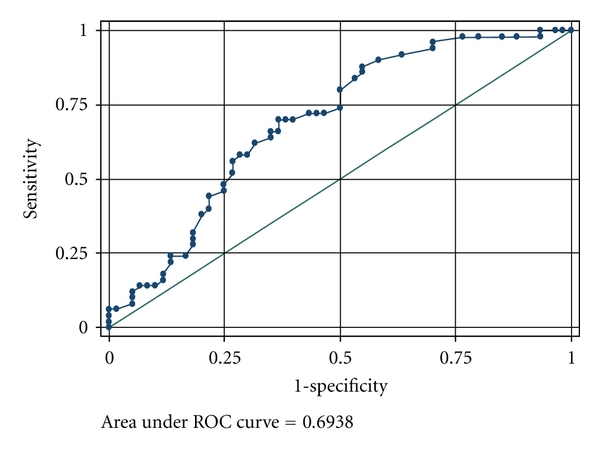
Receiver operating characteristics (ROCs) curve assessing the discriminatory ability of the MSKCC nomogram.

**Figure 2:**
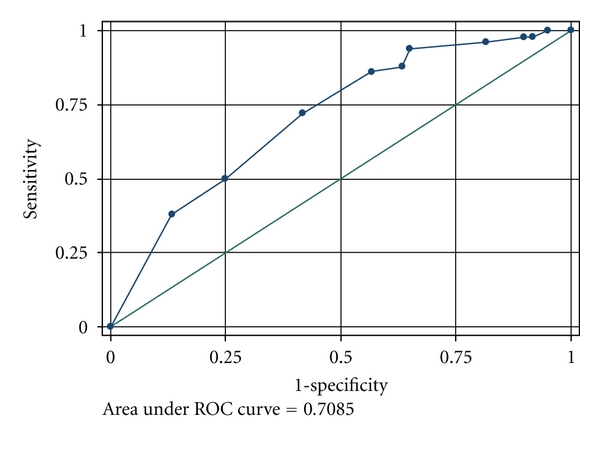
Receiver operating characteristics (ROC) curve assessing the discriminatory ability of the Tenon score.

The MSKCC nomogram and the Tenon score were validated in our study population. Based on the MSKCC nomogram, the median calculated probability in the group with SLN involvement alone was 19.5%, significantly lower than the calculated probability of 41.0% in the group with additional non-SLN involvement (*P* < 0.001) (Table 1). The discriminatory ability, as calculated from the area under the receiver operating curve (ROC) (AUC), was 0.69 (Figure 1). Similarly, the Tenon score also differentiated the group with SLN involvement alone from the group with additional non-SLN involvement (median score of 5.0 and 5.75 resp., *P* < 0.001), with an AUC of 0.71 (Figure 2). 

The subgroup of 15 patients (13.6%) with micrometastasis was further evaluated. Median tumour size was 20 mm (12 mm to 40 mm), median tumour grade was 2, and lymphovascular invasion was present in 10 patients (66.7%). Thirteen tumours were classified as invasive ductal carcinoma, 1 as invasive lobular carcinoma, and 1 as mucinous carcinoma. Calculated median Tenon score was 3.5 (1.5 to 5.0), and median predicted probability based on the MSKCC nomogram was 18% (7 to 51%). Only 1 patient (with a 25 mm invasive lobular carcinoma) had additional non-SLN involvement; calculated Tenon score was 5.0, and MSKCC predicted probability of non-SLN involvement was 18%.

## 4. Discussion

Involvement of the SLN raises the possibility of tumour spread to the rest of the axillary nodes. Current guidelines therefore recommend ALND whenever the SLN is involved by tumour, including by micrometastasis. The rationale for this is implied from previous experience with breast conserving surgery where it was shown that optimal postoperative irradiation did not reduce the risk of local recurrence if the surgical margins were inadequate, implying that residual disease may not be completely eradicated by adjuvant treatment. The Z0011 trial, however, reported results to the contrary. This study found no increase in recurrence nor any survival disadvantage in women with a positive SLN who did not undergo completion ALND, suggesting that any residual disease in the non-SLNs nodes may be effectively eradicated by adjuvant radiation and chemotherapy [6, 7]. It should, however, be noted that subjects included in the Z0011 study were a highly selected group, having only limited nodal disease, postoperative chemotherapy and chest wall irradiation which included the level I and II nodal basins. It is, therefore, too premature to conclude that it is safe to omit ALND in all patients with a positive SLN.

ALND is theoretically unnecessary when there is no additional involvement of the non-SLN nodes. In our study, 55% of patients with a positive SLN had no involvement of additional non-SLNs, in agreement with reports in the literature [16]. Although several models have been developed to predict the likelihood of non-SLN involvement, none have gained acceptance into routine practice. We have chosen to validate the MSKCC nomogram and the Tenon score as they were found to outperform other available models [15]. Both models are easy to use, the Tenon score being based on the sum of 3 variables to give a total score of 7, and an online calculator being available for the MSKCC nomogram. Both the MSKCC nomogram and Tenon score performed more poorly in our study population as compared to previous reports in Western populations, with an AUC of 0.69 and 0.71 respectively [8, 9, 17–19]. Although patients with SLN involvement alone had a significantly lower Tenon score as compared to those with additional non-SLN involvement, the median score of 5.0 was higher than the proposed threshold. Barranger and colleagues proposed a threshold of 3.5 since scores of 3.5 or less were associated with a 97.3% likelihood of having no additional non-SLN metastases [12]. If this threshold had been applied, only 24 patients in our study population would have avoided ALND. The median MSKCC probability among those with SLN involvement alone was 19.5%. Similarly, if the proposed threshold of 10% was taken, only 15 patients would have avoided ALND [8]. These findings suggest that a higher threshold may be necessary for Asian patients and may explain why both models performed more poorly in our study population. On the other hand, only 1 of the 11 patients who underwent a second surgery for ALND after metastatic deposits were found on the examination of the permanent sections had additional non-SLN involvement. This patient had a Tenon score of 5.0 and a predicted probability of 18% on the MSKCC nomogram. Although higher than the respective proposed thresholds for both models, both scores still fall within the median scores of those with SLN involvement alone in our study. Further studies in a larger study population will be needed to define an appropriate threshold for our population.

In our current practice, the decision ALND is made intraoperatively based on results of frozen section analysis of the SLN. This limits the types of variables that can be used to predict non-SLN involvement. Currently available models, including the MSKCC nomogram and Tenon Score, include variables which require analysis of the permanent formalin-fixed paraffin-embedded sections and can only be calculated postoperatively. The ratio of positive SLNs in relation to the total number of SLNs harvested has emerged as an independent predictor of non-SLN involvement in our study. This ratio was also found to be significant in both the MSKCC and Tenon models. In our study, 57% of patients with a positive SLN to total SLN ratio of 1 were found to have additional non-SLN metastases, as compared to 28% of those with a ratio of less than 0.5. The reliability of this ratio depends largely on the accuracy of SLN identification and intraoperative frozen section analysis. In our practice, the majority of SLN biopsy is performed with blue dye alone. Our results (SLN nonidentification rate of 2.6% and a false negative rate of 4.5%) are comparable to accepted standards (unpublished manuscript) [2, 4]. A possible criticism is that fewer SLNs are identified using blue dye alone. However, the median number of SLNs harvested per patient in our study was similar to that harvested in the study population from which the Tenon score was derived, where both blue dye and radiocolloid were used in combination [12]. Although we found the total number of SLNs harvested to be inversely correlated with the likelihood of non-SLN involvement, we failed to define an optimal number of SLNs that should be harvested. The median number of SLNs harvested in our patients was 2, occurring in 68 patients (62%). Although non-SLN involvement appeared more likely in patients when less than 2 SLNs were harvested, this did not reach statistical significance. Some studies have suggested that 3 is the optimal number of SLNs that should be harvested; fewer SLNs carry the risk of understaging, while the examination of more than 3 nodes does not increase sensitivity [20, 21]. Our sample size is likely too small for a significant correlation with the total number of SLNs harvested to be observed. We routinely perform intraoperative frozen section SLN analysis, with a false negative rate of 16%, often resulting from micrometastasis being found in deeper layers of the permanent sections (unpublished manuscript). 

The group of 15 patients with micrometastasis in the SLN is of particular interest. Current guidelines recommend ALND when the SLN is involved by micrometastasis since the possibility of non-SLN involvement cannot be ignored [22]. On the other hand, we have observed that micrometastasis alone predict for a low likelihood of additional non-SLN involvement; only 1 of the 15 patients was found to have non-SLN involvement. Several authors have proposed that a subgroup of patients with micrometastasis have such a negligible risk of non-SLN involvement that completion ALND may not be necessary; this group includes patients with tumour size of less than 10 mm, or less than 20 mm if of tubular, colloid, or medullary histology and micrometastatic deposits less than 1 mm in size [3, 22–25]. Of note is that more than 70% (11 of 15) of the patients with micrometastasis in our study had a Tenon score of 3.5 or less, suggesting that they might have avoided ALND if the threshold of 3.5 had been applied. Only 3 patients had a predicted MSKCC probability of less than 10%. It would seem that the Tenon score performs better, although both models have been reported to perform equally well in patients with micrometastasis [15, 26]. However, our study population is too small to allow us to draw any firm conclusions.

## 5. Conclusion

Although both the MSKCC nomogram and Tenon score differentiated between patients with SLN involvement only and those with non-SLN involvement, they performed less well than previously reported. It is possible that the proposed threshold for both models may need to be adjusted for Asian populations. The number of positive SLNs, the total number of SLNs harvested, and the size of the tumour deposits within the SLN were found to be independent predictors of non-SLN involvement. These variables are particularly relevant to our current practice where the decision for ALND is based on intraoperative frozen section SLN analysis. Further studies to evaluate the predictive potential of these factors will no doubt be useful in reducing the rate of unnecessary ALND in those with a negligible likelihood of non-SLN involvement.

## Figures and Tables

**Table 1:** Correlation analyses of SLN involvement and clinicopathological parameters (*n* = 110).

	Patients with SLN positive only (*n* = 60)	Patients with non-SLN involvement (*n* = 50)	*P* value
Median age (years)	54.0 (37.0 to 80.0)	51.5 (30.0 to 77.0)	0.42
Ethnicity			0.70
Chinese	47	36	
Malay	8	8	
Indian	2	4	
Others	3	2	
Family history of breast cancer			0.53
Yes	7	8	
No	52	42	
Tumour histology			0.20
IDC	56	42	
ILC	4	7	
Median tumour size (mm)	20.0 (4.0 to 80.0)	24.5 (1.0 t o 55.0)	0.03
Tumour grade			0.99
1	5	6	
2	28	19	
3	26	23	
Lymphovascular invasion			0.81
Present	26	22	
Absent	29	27	
Associated DCIS			0.97
EIC	10	8	
DCIS	25	19	
None	22	17	
Oestrogen receptor status			0.84
Positive	42	35	
Negative	17	13	
Progesterone receptor status			0.42
Positive	31	21	
Negative	28	26	
HER2 status			0.16
Positive	16	10	
Negative	27	33	
Size of nodal disease			0.01
Macrometastases	46	49	
Micrometastases	14	1	
Ratio of positive SLN to total SLN harvested			0.01
<0.5	20	8	
0.5 to 1	14	8	
1	26	34	
Number of axillary nodes			0.03
<10	11	3	
10 to 20	25	16	
20 to 30	17	24	
>30	7	7	
Distant recurrence			0.34
Yes	5	7	
No	55	43	
Median Tenon score	5.0 (1.5 to 7.0)	5.75 (2 to 7)	<0.001
Median MSKCC probability	19.5 (3.0 to 74.0)	41.0 (6.0 to 89.0)	<0.001

**Table 2:** Multivariate analysis Cox regression model for non-SLN involvement for standard clinicopathological parameters (*n* = 110).

	Odds ratio	*P* value	95% confidence interval
Number of positive SLNs	2.05	0.04	1.04–4.01
Total number of SLNs harvested	0.73	0.03	0.55–0.96
Micrometastasis	0.06	0.01	0.01–0.54
Tumour size	1.03	0.14	0.99–1.07
Total number of axillary LN harvested	1.06	0.03	1.01–1.12
